# Prevalence and influencing factors of sleep disorders among preschool children in Urumqi city: a cross-sectional survey

**DOI:** 10.1186/s13052-023-01477-w

**Published:** 2023-06-07

**Authors:** Yongwei Gao, Peiru Xu, Maiming Aizetiguli, Shan Surong, Zhaoxuan Zhu, Jing Zhang

**Affiliations:** College of Pediatrics, Xinjiang Medical University, Urumqi, Xinjiang China

**Keywords:** Sleep disorders, Influencing factors, Preschool children, Prevalence, Urumqi

## Abstract

**Background:**

Sleep disorders refer to physiological and psychological states that cause adverse consequences due to the inability to fall asleep or poor sleep quality. The prevalence of sleep disorders varies greatly in different countries and regions due to different causes. This study aimed to investigate the prevalence and influencing factors of sleep disorders among preschool children in Urumqi city, China.

**Methods:**

A cross-sectional study was conducted with stratified random cluster sampling. Children aged 3–6 years old in one kindergarten randomly selected from each of the 8 districts of Urumqi from March to July 2022, and their parents were surveyed with a sleep quality questionnaire.

**Results:**

The prevalence of sleep disorders among preschool children in Urumqi was 14.29% (191/1336), and the prevalence of different symptoms was 42.81% for limb movements, 19.61% for snoring, 18.11% for bruxism, 16.39% for sleep talking, 12.57% for sweating, 11.60% for nocturnal awakening, 8.46% for nightmares, 6.89% for bed wetting, 3.74% for apnea, and 3.29% for sleepwalking. The prevalence of body movements, snoring, sweating, night-wake, nightmares, bed-wetting, apnea, and sleepwalking among different ethnicities were significantly different (P < 0.05). Multivariate analysis revealed that the major risk factors of sleep disorders were difficulty adapting to new environments, unwillingness to express emotions, inconsistent attitudes of the family toward children’s education, running before bedtime, strict family education methods, etc.

**Conclusion:**

The prevalence of sleep disorders in preschool children in Urumqi is lower than the average level reported in other studies. Many factors affect the prevalence of sleep disorders in preschool children, but it is necessary to focus on the ability to adapt to new environments, psychological problems, and the impact of family education on sleep disorders. Further studies on the prevention and treatment of sleep disorders are needed for different ethnicities.

## Background

Sleep disorders defined as conditions that impairs a person’s sleep and prevents their restful sleep [1]. These disorders can impact a child’s physical, neurological, and linguistic development as well as mental health, cognitive, emotional, social well-being and behavioral well-being [2–8]. Sleep disorders among children are a significant public health concern worldwide, with a rising prevalence among preschool children in various countries. The prevalence of sleep disorders varies widely across different regions due to various factors [7]. Research indicates that the prevalence of sleep disorders among preschool children is significantly higher in China than in European and American countries [9]. The preschool years are a critical period of rapid physical and mental development and a time when sleep problems often emerge and become frequent. Studies show that up to 40% of preschool children are affected by sleep disorders [10]. Urumqi is a multi-ethnic city with individuals having varied genetic characteristics, lifestyles, and dietary habits. Despite this, there is still a lack of sleep data for preschool children in Urumqi. To address this knowledge gap, a questionnaire-based was conducted to determine the prevalence and influencing factors of sleep disorders among preschool children in Urumqi. The results of this study will provide a basis for preventing and.

treating sleep disorders in preschool children.

## Methods

### Research object

This study utilized a cross-sectional design with stratified random cluster sampling to investigate sleep disorders among preschool children in Urumqi. Between March 2022 to July 2022, one kindergarten was randomly selected from 8 districts of Urumqi. Children aged 3–6 years from each kindergarten were included in this study if they met the following criteria: (1) Age 3–6 years; (2) Resided in Urumqi for more than 1 year, and parents or guardians were willing to participate in the survey. (3)A questionnaire was administered to the study population who met the inclusion criteria.

### Questionnaire design

The questionnaire was designed based on the children’s sleep habits questionnaire (CSHQ) recommended in the Sleep Guide for Children Aged 0–5 years issued by the National Health and Family Planning Commission of China in 2017 [11]. The questionnaire was prepared in combination with similar domestic research experience and Urumqi special points, including the basic situation of children, family environment, previous disease history, personal life habits, sleep habits, etc. It consisted of 33 items and was used to evaluate the frequency of each item over the past month. According to the frequency described by the guardian, each item was scored from 1 to 3, with higher scores indicating more sleep problems. A total score of > 54 points was considered indicative of a sleep disorder. The Chinese preschool children refer to children aged 3–6 years and therefore, the diagnostic criteria in this study for sleep disorders in children aged 6 years were same as those in children aged 3–5 years. To ensure the reliability and validity of the questionnaire, Cronbach α coefficient was calculated and found to be 0.71.

### Investigation method

After parent-teacher conferences meeting, parents or guardians familiar with the children’s sleep habits answered the questionnaire on their Mobile phones or computers based on their children’s sleep habits over the past month, and “Questionnaire Star” collected the results and eliminated incomplete questionnaires.

### Statistics

Statistical analysis was carried out using SPSS26.0. For descriptive statistics, count data were expressed as frequencies (percentages), and measurement data were expressed by the mean and standard deviation .The t-test was used for two-way comparison when the measurement data conformed to a normal distribution, and F-test was used for multi-group comparison. The rank sum test was used for two-way comparisons when the data did not conform to a normal distribution, and the H test was used for multiple-group comparisons. The chi-square test was used for comparing count data. One-way analysis was used to identify factors related to sleep disorders affecting children, and variables with significant differences (P < 0.05) were subjected to logistic regression analysis, and P < 0.05 was considered statistically significant.

## Results

### Basic information about the study subjects

A total of 1550 children were investigated in this study, out of which 1336 preschool children were surveyed, yielding a response rate of 86.19%. The child cohort comprised 691 (51.72%) boys and 645 (48.28%) girls. The age of participants was between 3 and 6 years (4.54 ± 1.06). There was 959 Han Chinese (71.78%), 231 Uyghurs (17.29%), 79 Hui (5.91%), 22 Kazakhs (1.65%), and 45 participants from other ethnicities (3.37%).

### Prevalence of sleep disorders among preschool children

Table 1 shows that 191 (14.29%) children with sleep disorders, including 93 cases (48.69%) of boys and 98 cases (51.31%) of girls; 29 cases (2.17%) at age 3, 54 cases (28.27%) at age 4, 55 cases (28.8%) at age 5, and 53 cases (27.75%) at age 6. A total of 127 cases (66.49%) of Han Chinese, 43 cases (22.51%) of Uyghur (22.51%), 12 cases (6.28%) of Hui, 4 cases (2.09%) of Kazakh, and 5 cases of other ethnicities (2.61%) were observed. No statistically significant difference was observed in the prevalence of sleep disorders among different genders, ethnicities, and ages (P > 0.05) .

**Table 1:** Prevalence of sleep disorders among preschool children

Projects		Total		Sleep disorders		Non-sleep disorders		P
		n	%	n	%	n	%	
Sex	Boys	691	51.72	93	13.46	598	86.54	0.365
	Girls	645	48.28	98	15.19	547	84.81	
Ethnicity	Han Chinese	959	71.78	127	13.24	832	86.76	0.279
	Uighur	231	17.29	43	18.61	188	81.39	
	Hui	79	5.91	12	15.19	67	84.81	
	Kazakh	22	1.65	4	18.18	18	81.82	
	Other Ethnicities	45	3.37	5	11.11	40	88.89	
Age	3~4	281	21.03	29	10.32	252	89.68	0.102
	4~5	361	27.02	54	14.96	307	85.04	
	5~6	390	29.19	55	14.10	335	85.90	
	6~7	304	22.75	53	17.43	251	82.57	

### Prevalence of different symptoms associated with sleep disorders among preschool children

Table 2 shows that the prevalence of symptoms associated with sleep disorders among preschool children was 42.81% for limb movements, 19.61% for snoring, 18.11% for bruxism, 16.39% for sleep talking, 12.57% for sweating, 11.60% for nocturnal awakening, 8.46% for nightmares, 6.89% for bed-wetting, 3.74% for apnea and 3.29% for sleepwalking, with no statistically significant difference between the above symptoms in boys and girls (p > 0.05). Statistically significant differences in limb movements, snoring, sweating, night waking, nightmares, bed wetting, apnea, and sleepwalking among the different ethnicities were also observed (P < 0.05). While Uyghur preschool children had the highest prevalence of sleepwalking, snoring, and apnea, Kazakh preschool children had the highest prevalence of limb movements and bed-wetting. Preschool children of other ethnicities had the highest prevalence of sweating, nightmares, and nocturnal waking (p < 0.05). Bruxism and sweating showed statistically significant differences between ages (p < 0.05).

**Table 2:** Prevalence of different symptoms associated with sleep disorders among preschool children n (%)

Projects	Cases	Sleep Walking	Bed Wetting	Sleep Talking	Limb Movements	Bruxism	Snoring	Sweating	Nightmare	Nocturnal Wakening	Apnea
Total	1336	44(3.29)	133 (9.96)	219(16.39)	572(42.81)	242(18.11)	262(19.61)	168(12.57)	113(8.46)	155(11.60)	50(3.74)
Gender											
Boys	691	18(2.60)	76(11.00)	107(15.48)	300(43.42)	137(19.83)	149(21.56)	84(12.16)	63(9.12)	74(10.71)	31(4.49)
Girls	645	26(4.03)	57(8.84)	112(17.36)	272(42.17)	105(16.28)	113(17.52)	84(13.02)	50(7.75)	81(12.56)	19(2.95)
P		0.144	0.187	0.354	0.646	0.093	0.063	0.633	0.370	0.292	0.138
Ethnicity											
Han Chinese	959	18(1.88)	87(9.07)	150(15.64)	426(44.42)	166(17.31)	146(15.22)	73(7.61)	50(5.21)	82(8.55)	28(2.92)
Uighur	231	21(9.09)	35(15.15)	48(20.78)	79(34.20)	52(22.51)	82(35.50)	64(27.71)	43(18.61)	40(17.32)	16(6.93)
Hui	79	3(3.80)	5(6.33)	13(16.46)	39(49.37)	10(12.66)	16(20.25)	13(16.46)	7(8.86)	15(18.99)	2(2.53)
Kazakh	22	1(4.55)	6(27.27)	4(18.18)	12(54.55)	7(31.82)	4(18.18)	3(13.64)	3(13.64)	3(13.64)	1(4.55)
Other Ethnicity	45	1(2.22)	0(0)	4(8.89)	16(35.56)	7(15.56)	14(31.11)	15(33.33)	10(22.22)	15(33.33)	3(6.67)
P		< 0.001^*^	< 0.001^*^	0.236	0.021^*^	0.092	< 0.001^*^	< 0.001^*^	< 0.001^*^	< 0.001^*^	0.045^*^
Age											
3~4	281	9(3.20)	28(9.96)	54(19.22)	129(45.91)	25(8.90)	50(17.79)	53(18.86)	26(9.25)	38(13.52)	6(2.14)
4~5	361	7(1.94)	33(9.14)	59(16.34)	160(44.32)	60(16.62)	80(22.16)	41(11.36)	25(6.93)	45(12.47)	14(3.88)
5~6	390	14(3.59)	35(8.97)	57(14.62)	165(42.31)	80(20.51)	67(17.18)	44(11.28)	30(7.69)	36(9.23)	14(3.59)
6~7	304	14(3.29)	37(12.17)	49(16.12)	118(38.82)	77(25.33)	65(21.38)	30(9.87)	32(10.53)	36(11.84)	16(5.26)
P		0.280	0.503	0.466	0.326	< 0.001^*^	0.246	0.004^*^	0.348	0.330	0.260

^*^P < 0.05

### Analysis of influencing factors of sleep disorders among preschool children

A one-way analysis was conducted on a case-by-case basis by using the presence of sleep disorders as the dependent variable Y, and various contributing factors of sleep disorders as the independent variable X. Results revealed that recurrent respiratory infections, age, BMI, impulsive and active, ability to adapt to new environments, willingness to express emotions, duration of outdoor activity, time spent on electronic gadgets, feeding patterns, feeding pets, noisy living room, eating before bedtime, home education attitudes and approach toward children, type of family, running before bedtime, maternal pregnancy or postpartum depression, mother having symptoms of sleep disorders, diet rules, anorexia, partial eating, maternal education background, maternal occupation, and total annual household income were the 24 statistically significant factors (p < 0.05). Logistic regression analysis carried out by introducing the variables that were correlated into the equation showed that 11 variables ended up in the logistic regression equation for sleep disorders among preschool children, ranked in order of their relative risk (Table 3).

**Table 3:** Logistic regression analysis of influencing factor of sleep disorders among preschool children

Factors	B	SE	Wald cardinality	P-value	OR	95% CI	
Constants	-1.052	0.835	1.586	0.208	0.349		
Does not adapt easily to new environments	0.724	0.219	10.953	0.001	2.062	1.343	3.165
Never willing to show emotions	0.931	0.426	4.774	0.029	2.537	1.101	5.848
Inconsistent attitudes toward teaching children	0.636	0.2	10.141	0.001	1.888	1.277	2.793
Running before bedtime	0.614	0.219	7.881	0.005	1.847	1.203	2.834
Irregular diet	0.6	0.264	5.147	0.023	1.822	1.085	3.059
Tough Discipline Homeschooling Style	0.529	0.243	4.735	0.03	1.697	1.054	2.732
No partial eating	-0.41	0.2	4.222	0.04	0.664	0.449	0.981
High total annual household income	-0.454	0.229	3.94	0.047	0.635	0.405	0.994
No maternal pregnancy or postnatal depression	-0.465	0.191	5.914	0.015	0.628	0.432	0.914
No food before bedtime	-0.59	0.19	9.641	0.002	0.554	0.382	0.804
No anorexia:	-0.813	0.2	16.464	< 0.001	0.443	0.299	0.657

B : Coefficient of regression; SE: Standard error; OR Odds ratio; CI: Confidence interval

## Discussion

Sleep involves physiological changes in several organ systems and plays a critical role in the growth and development of infants, children, and adolescents [12]. High-quality and sufficient sleep is essential for healthy child development [13]. Studies have shown an association between sleep disorders and long-term adverse childhood outcomes focusing on persistence of sleep disorders beyond 2–5 years as the measure of interest [14]. Early childhood sleep is influenced by social and biological factors that differ from those that affect sleep in adolescence and adulthood [15].

The present study examined the prevalence of sleep disorders in preschool children in Urumqi and found it to be 14.29%, the findings were similar to Korea (15%) [10], lower compared to Turkey (35.8%) [16], a meta-analysis of sleep disorders among children in mainland China (38.9%) [17], and other studies conducted abroad (20–25%) [18]. The difference in findings may be related to regional variations in sleep disorders and the different assessment tools used.

Studies among school-aged children show sleep disorders is often more prevalent among racial minority groups, race is an important factor influencing sleep patterns in the preschool children [19]. There were significant differences in sleep disorders among Jewish and Muslim children in Israel, and cultural sleep practices may contribute to the differences [20]. There were significant differences between gender in relation to being at high risk of developing sleep disorders [21].A meta-analysis revealed a higher prevalence of sleep disorders among boys than girls, and the prevalence of sleep disorders among children increased with age [22]. This study found no statistically significant differences in the prevalence of sleep disorders by gender, age, and ethnicity among preschool children.

A meta-analysis revealed that the prevalence of sweating and limb movements was significantly higher than other symptoms of sleep disorders [22]. This study found the prevalence of limb movements, snoring, bruxism, sleep talking, sweating, nocturnal awakening, nightmares, bed wetting, apnea, and sleepwalking, with no statistically significant differences between preschool boys and girls. There are hardly any studies in the national and international literature assessing the differences in the prevalence of sleep disorders symptoms among preschool children by ethnicity. The present study found statistically significant differences in the prevalence of limb movements, snoring, sweating, sleepwalking, nightmares, bed wetting, apnea, and sleepwalking among different different ethnicities. Further research is necessary to better understand the prevention and treatment of sleep disorders in different ethnicities.

This study also identified several factors that influenced the prevalence of sleep disorders among preschool children, including difficulty adapting to a new environment, reluctance to show emotion, inconsistent attitudes toward family education, running before bedtime, and strict discipline of family education. There are complex and bidirectional associations between sleep and mental health [23]. Sleep disorders are also related to neurodevelopmental disorders, depressive disorders, anxiety disorders, disruptive, conduct disorders, emotions [24–29]. It is increasingly well-established that sleep is linked to mental health, but the mechanism is unclear.

The period between 0 and 6 years is a critical period for the formation of social adaptability. Family is the most important environmental factor affecting the development of early social adaptability in children. Adverse parenting styles (e.g., low positivity and high negativity) have been found to be associated with low sleep quality, negative mood, daytime sleepiness, and anxiety/ depression symptoms among adolescents [30]. Whereas positive parenting behaviors seem to promote good sleep behaviors and consequently reduce the risk for problematic behaviors among adolescents [31]. The early comprehensive intervention of OSAHS Children with OSAHS can improve their social adjustment. Inconsistent parenting attitudes at home can have a detrimental effect on children’s mental health [32], leading to anxiety, which can have an impact on their sleep. Proper bedtime and mood management can aid in reducing sleep disorders [33], and bedtime running can show excitement and other strong mood swings that can potentially affect the sleep of children. Inappropriate parenting styles can severely affect the mental health of children in their early developmental years and have severe effects later in the child’s growth and development [34]. Parental coercion or hostility during the preschool years can lead to internal stress, dysregulation of the body’s regulatory system, hormonal imbalances, and disruption of sleep quality [35]. The results suggest that attention to the adaptability of preschool children in new environments, psychological problems, and family education has the potential to reduce the prevalence of sleep disorders and contribute positively to their healthy physical and mental development.

The sleep disorders is reported by the parents, and some reference to any cultural factors that could be decisive in the perception of the disorder by the parents. The perception by parents of a child sleep disorders should be influenced by specific levels of tolerability that could depend on cultural, educational and social characteristics. Waddington H et al. found that lower paternal education and lower family income were related to increased sleep disorders [36]. Shervin A et al. found that parental education was associated with children’s sleep problems [37]. This study found that paternal education and occupation were not risk factors for sleep disorders in preschool children, and that high total annual family income was a protective factor for sleep disorders in preschool children.

## Limitations

The study selected preschool children in Urumqi, and therefore, the findings cannot be extrapolated for children in different geographical areas. Also, the data was obtained based on parents’ recollections, leading to the possibility of recall bias.

## Conclusion

In conclusion, the prevalence of sleep disorders among preschool children in Urumqi was lower than the average level reported in previous studies conducted both in China and internationally. The study highlights the significance of various factors that affect the prevalence of sleep disorders among preschool children, including environmental adaptability, psychological factors, and family education. Therefore, it is important for all sectors of society to pay attention to these factors and to conduct further in-depth research on the prevention and treatment of sleep disorders in preschool children, particularly for different ethnicities.

## Data Availability

The datasets used and/or analyzed during the current study are available from the corresponding author on reasonable request.
